# Accountability in the NHS: the impact on cancer care

**DOI:** 10.3332/ecancer.2018.ed83

**Published:** 2018-07-18

**Authors:** Aislinn Macklin-Doherty

**Affiliations:** The Institute of Cancer Research, Cotswold Road, Sutton, SM2 5PT, UK

**Keywords:** NHS, NHS accountability, cancer care, oncology, cancer inequality, healthcare delivery, healthcare models, NHS transparency

## Abstract

Accountability of service delivery is becoming increasingly complex and never has this been more apparent than in the field of Oncology. Cancer care has an unrivalled level of complexity not only in the heterogeneity of management of the disease itself but increasingly in the myriad of service providers, specialities, policymakers and regulatory bodies overseeing its delivery. The stepwise series of changes to NHS structures over recent decades has had an enormous impact on our ability to answer key questions which lie at the heart of accountability: who is making the key decisions about changes to cancer care delivery? What are these reforms achieving? How can they be influenced?

It is only through clear and transparent decision-making that we may have any hope of implementing, monitoring and influencing the effects of evidence-based change.

However, with growing complexity of service structures and an increasing number of bodies developing ambitious and complex strategies, in a context of resource restraint and system pressures, it has become very difficult to answer these questions clearly. This increasing lack of clarity and transparency around such fundamental questions may mean that, despite there being such a pressing need and apparent desire for accountability in cancer care, paradoxically we may actually be deviating further and further away from this. Perhaps it is time for less complexity and for the decision-makers to get back to some fundamental principles which clinicians have embraced in evidence-based medicine: what is being done and by whom? Is this change beneficial and if not how can we influence change?

## Introduction

Cancer care is a national priority and resources are ring-fenced and significant investment is made accordingly. [1] With UK cancer incidence continuing to rise, [2] there is a simultaneous increasing proportion of 10-year cancer survivors, [3] and with rapidly advancing diagnostic and treatment technologies, the demand to improve and expand services for more patients who are living longer will also rise. [4]

As the service has expanded, so too has the complexity of the system of delivery itself. This is epitomised in the field of oncology. The way in which we deliver cancer care in the UK has been subject to huge reorganisation over the last 3 decades.[1, 6] In the early 1990s it became apparent that there were significant deficiencies and variability in cancer care delivery and patient outcomes both within the UK and in comparison to our European counterparts.[7, 8] This led to some extremely ambitious cancer-specific initiatives and system reforms which have paved the way for similar models of reform in other specialties. [1, 9-11] In parallel, there have been numerous structural and legislative reorganisations of the wider NHS within which these initiatives have been implemented, which have led to the creation of a myriad of bodies now involved in purchasing, commissioning, delivering and regulating cancer care delivery. [5] The question therefore of who is “accountable” for decision-making for healthcare delivery in oncology has never been so complex to unpick and yet so fundamental to address.

Accountability in how we deliver care should not be a theoretical aim outlined within strategy documents but must be clearly evident in all of the decisions made. Without accountability it is very difficult to measure what has been done, by whom, how and why. Accountability in care delivery must be strived towards by policymakers and politicians in equivalence to how clinicians follow a model of evidence-based medicine. Otherwise it becomes very difficult to unpick what has changed, the impact of the change and perhaps more importantly how to adapt to changes that are not working. This editorial attempts to explore current accountability in cancer care by asking three fundamental questions:

1. How has cancer care been reformed and who is now making the key decisions about care delivery?
2. Are these reforms working?
3. How can these policies be influenced?

## 1. How has cancer care been reformed and who is now making the key decisions about cancer care delivery?

In 2012, one of the most significant “top down” reorganisations in NHS history was enacted, the Health and Social Care Act 2012. [12] This followed two decades of a series of controversial market-style reforms to de-centralise control of delivery of services and move over to a competitive purchaser-provider system. [13] These changes have led to a multiplication of the number of bodies involved in overseeing, planning and implement delivery of cancer care and now, in addition, new bodies which regulate it. [12] This move to “devolve” and de-centralise certain aspects of decision-making led to the dissolution of cancer-specific national infrastructure such as the National Cancer Action Team. Currently the most influential national bodies which commission the mainstay of cancer treatments (surgery, chemotherapy and radiotherapy), screening and prevention are the arms-length bodies, NHS England and Public Health England. [14] These are closely related to the Department of Health and answerable to Government through contractual obligations and yet are designed to simultaneously have decision-making autonomy from Government. This new dynamic has added an inevitable layer of complexity and confusion as to where responsibility lies. [12] The roles and responsibilities of these new NHS organisations have not generally been well understood and widespread concerns have been raised by a range of cancer professionals from GPs to policymakers and commissioners about fragmentation and a “vacuum” of national aims and cohesion [15] despite stated aims being to make care more accountable, democratically legitimate and patient-centred. [16]

In addition to these new bodies, primary care trusts which previously oversaw spending of 85% of the NHS budgets were abolished and in their place, regionally-based clinical commissioning groups (CCGs) were given the responsibility of planning and overseeing how the majority (approximately two thirds) of NHS services are delivered. These are monitored by NHS England through outcomes frameworks drawn up centrally and by an additional arm’s length financial regulatory body, Monitor. [12]

**Figure 1.**
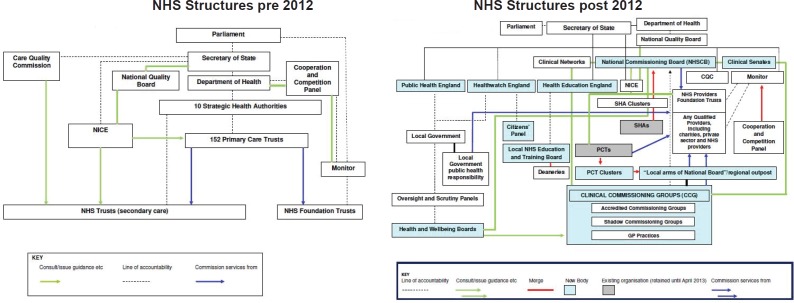
The NHS structures in England pre and post the Health and Social Care Act [60].

CCGs are loosely defined and can involve a range of members including GPs, other local clinicians, managerial and financial staff, private sector, charity and lay representatives. Although CCGs were premised on the idea that clinicians would be put at the heart of clinical decision-making, a number of studies have demonstrated huge variation in how they are made up and the roles played by those within. [17, 18] A significant number of those who are on CCG boards have financial conflicts of interest, commissioning services from providers they are associated with, [19] and ultimately this has led to fragmentation and even greater complexity of service delivery with lack of clarity and consistency in who determines how services are delivered. [12, 20] The nature of this complexity is well illustrated by juxtaposing the structures that were in existence in the NHS prior to the Health and Social Care Act 2012 and to those introduced following it outlined in Figure 1 in 2013:

Alongside these wider huge changes to how the NHS is run generally, a series of ambitious programmes of change relating specifically to how cancer services are configured were also implemented over roughly the same period. The largest was triggered following the landmark Calman-Hine Report of 1995. [6] This was written in light of growing concerns about a healthcare “lottery” emerging in the UK [8, 22] with a lack of a standardised approach to diagnosis and treatment of a highly complex disease with emerging evidence of the UK’s survival outcome “gap” [23] compared to other European countries. Nationwide reforms were premised on the idea of streamlining services and prioritising “patient-centred care” which commenced with the National Cancer Plan in 2000 [9] followed by the National Cancer Reform Strategy in 2007. [10] Each has set out to improve cancer survival rates in the UK to close the gap with other comparable countries’ survival rates, and to reduce inequalities related to cancer survival. The most recent far-reaching plans have been put forward by the independent cancer taskforce who in 2015 proposed NHS England’s National Cancer Strategy: Achieving World Class Outcomes. [1] This group have initiated a series of further radical changes to models of cancer care delivery in the last 2 years, with the stated aims of modernising and transforming services to improve and speed up early diagnosis, improve patient experience and quality of life, reduce inequalities of accessibility to care and cancer survival across the country, whilst making care more integrated and streamlined.

The answer therefore to who precisely is making the decisions about cancer care delivery is: it’s complicated. Cancer care clearly represents one of the most complex specialities in terms of service delivery. The heterogeneity in disease behaviour between and within tumour types and patients combined with the multiple interacting specialties, settings, technologies and disciplines means achieving streamlined, effective care has enormous challenges. Prevention, diagnosis, multidisciplinary treatments, supportive and palliative care and now increasingly survivorship care need to all be provided for in an integrated system which allows for efficient and seamless management of patients. However, the increasing complexity of commissioning and managing these services seems to be overtaking the complexity of the science of the care itself and what is needed. With every new initiative, strategy for reform or system overhaul we seem to move further away from being able to answer vital questions on who is responsible for changing and overseeing care delivery. Many clinicians, NHS managers and NHS leaders themselves are increasingly confused about who is responsible for delivery of cancer services at a local and national level [2, 24] which is one of the most fundamental markers of the state of accountability and is what the Department of Health have stated they are trying to achieve. [12].

The system of delivery seems to have become a very confusing, complicated and variable web of structures depending on the specific area of the country, tumour type and aspect of service provision one is focussing on; with those responsible for overseeing changes to care and the care itself ranging from a mixture of GPs and hospital clinicians who are able to engage with the process of commissioning and service delivery alongside a clinical career, finance managers, the private and third sector, lay representatives and politicians. [25]

## 2. Are these reforms working?

The progress made towards achieving the ambitious goals set out within these reforms unfortunately seems somewhat lacking when looking at the real-world measured clinical outcomes. After nearly 20 years of enormous shake-ups in provision, evidence is now emerging that the impact on one of the key aims of these reorganisations, reducing inequalities of cancer outcomes and significantly improving survival rates, has not only been disappointingly absent, but there is evidence of a persistent and even growing survival “gap” both across the UK and across Europe in a number of tumour types. [5, 26] The reasons for this are varied depending on the tumour type but have been extensively analysed [27-29] and the “avoidable” deaths from cancer are largely attributed to delays in diagnosis and varying access to high quality treatments and care early on in the disease. [30] Survival and mortality rates are also persistently and significantly correlated with socioeconomic status. [31]

This lack of progress in relation to outcomes seems somewhat at odds with the reportedly groundbreaking increase in investment in new drugs, additional staff and equipment [32] with a (inflation-adjusted) 35% increase in annual expenditure on cancer services between 2001-2004. [5]

However, what is also emerging through these heterogeneous outcomes is a picture that reflects a wide geographical variation in the proportion of cancers picked up at an early stage, an important surrogate correlating with survival and mortality. [33] Heterogeneous uptake and access to screening and diagnostic procedures for certain tumour types, variability in use of referral pathways and differing patient behaviours are all implicated in these differences across the UK. [33] Without a unified national infrastructure to address these variations it appears as though periodic “injections” of investment across the country appear sporadic and reactionary to fluctuating regional demands without a coherent and joined up rationale which would address these inequalities. Many of the most recent announcements of investment have been through the controversial “Sustainability and Transformation Plans” [34] which have been criticised for propagating further fragmentation and inequality in service provision nationally. [35,36]

Whilst there has been a great deal of investment focussed within centralised large “Vanguard” centres at the forefront of driving through these next big reforms in cancer care delivery, [37] the concern is that this lack of uniformity of investment with simultaneous constriction of overall budgets could be leading to rationing of care in other areas [38-40] and contributing to this stall of improvement in more equitable outcomes.

An important contextual factor contributing to this has been the wider pressures that the NHS has been facing in recent years and the difficulty of achieving such ambitious goals within a context of increasing resource restraint is becoming all too apparent.

**Figure 2.**
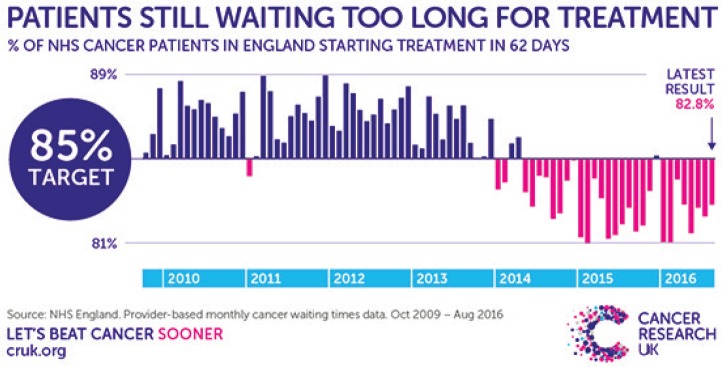
Proportion of patients starting treatment within 62-day target 2009-2016 [44].

In January 2018 calls from NHS England to cancel elective surgeries and outpatient appointments in order to cope with acute healthcare demands were unprecedented [41] and included a significant number of cancelled cancer operations. [42] The 62-day target of referral for suspected cancer to first definitive cancer treatment, implemented in 2000 to address the problem of lengthy cancer treatment delays in the UK, has been increasingly missed over the last 4-5 years [43] with a worrying ongoing downward trend as highlighted in Figure 2: [44]

Pressures on the workforce and dramatically reduced inpatient bed numbers [45, 46] have undoubtedly contributed to the challenges. Shortfalls in clinical staff numbers are now measuring in the tens of thousands according to figures from Health Education England with the ratio of doctors and nursing staff to patients falling behind most other European countries. [47] Almost all major treating oncology specialties and oncology healthcare professionals [48, 49] have put out calls to drastically increase their practising numbers, emphasising that this pressure is being felt acutely across oncology. The issue is so pressing a national workforce strategy drawn up to tackle this. [50]

Therefore within this overall context of pressured budgets and constrained, unequally distributed resources, [51] the variability in levels of provision may well be what is being reflected in the lack of improvement in addressing the stated goals of these strategies. [52] More concerning is that this is actually having a negative impacting on cancer survival. [40]

It is therefore important to raise the question of whether the reality of these proposals align with NHS principles and the stated aims of the reforms or are in fact progressing towards a deviation away from them.

## 3. How can key decisions be influenced?

In light of the increasing complexity of service delivery, the vast range of people and disciplines involved in decision-making with ambitious strategic changes, yet lack of consistent evidence of population benefit, this question becomes absolutely vital.

The NHS is undergoing enormous changes at a time of significant strain and often the majority of clinicians feel they are unable to keep up with the myriad changes and as a result do not feel involved or consulted. [53, 54] Lack of clinical involvement in key decision-making is worrying when it is clinicians who will be delivering much of the frontline care and when their involvement is consistently correlated with better outcomes for patients. [9, 55]

Patient and public involvement has also been largely absent from big service changes designed to drive through huge structural changes in how cancer care is delivered. [56] This is either due to a lack of active consultation and plans being unpublished or the speed of change being so quick it has not allowed for public involvement. Media coverage informing the public of these changes has also been sparse. A lack of parliamentary scrutiny on many of these large structural changes has also been raised as lacking, so much so that NHS England now faces a legal challenge from academics and campaign groups [57] with concerns raised that these changes may compromise the principles of equitable and high quality care delivery for the population. [58] The question of accountability is raised in the very first line of the NHS constitution, the premise on which the UK health service was created in 1948. It states that “The NHS belongs to the people.” [59]

As the NHS is publicly owned, the responsibility of delivering healthcare according to the key agreed principles is therefore ultimately answerable to and must be accountable to the public.

Again, with the vast number of ongoing changes and ever-increasing complexity of services, the importance of remembering the core principles lying at the heart of NHS cancer care being accountable to the public, this needs to be urgently addressed.

## Conclusions

Accountability in how we deliver cancer care has been frequently stated as a fundamental objective in Government strategy and policy documents over many years. It is increasingly and more urgently being advocated for by a number of professional bodies, policymakers and organisations involved in the delivery of cancer care.

However, there appears to be a notable difference between the laudably stated aims ascribed to service delivery change, and the reality of what has actually been achieved and what is currently being achieved for cancer patients.

The era of evidence-based medicine has seen the advancement of transparency, measurability, reproducibility and standardisation in how we clinically and biologically approach treating patients. Never has this been so important as in a highly complex disease such as cancer.

However, the structuring and investment in cancer care delivery seems to have fallen short of comparable standards. By a wide range of indicators, the vast plans put in place over many decades to improve and tackle deficiencies in UK cancer care, do not seem to have achieved what they set out to do in improving comprehensive, equitable and high quality cancer care. Meanwhile the delivery of services becomes ever more piecemeal, confusing and complex.

Accountability must be the reality lying at the heart of all system-wide changes so that we can apply the same high standards seen in evidence-based medicine to service delivery change. Who is making the key decisions about changes to cancer care delivery? What are these reforms achieving? How can they be influenced? These are the key questions about care delivery which should be easy to respond to if we truly had accountability at every level of care delivery but which, as has been highlighted here, have paradoxically in recent decades with each system overhaul become frustratingly even more difficult to answer. Perhaps it is time to drastically reduce this complexity in care delivery and reform, streamline and nationally coordinate cancer care and get back to basic principles with transparent models of care delivery. Only then might it be possible to start to be able to answer these questions and meaningfully tackle what was set out to be addressed over two decades ago.
